# Vascular Calcification in Chronic Kidney Disease: Diversity in the Vessel Wall

**DOI:** 10.3390/biomedicines9040404

**Published:** 2021-04-08

**Authors:** Prabhatchandra Dube, Armelle DeRiso, Mitra Patel, Dhanushya Battepati, Bella Khatib-Shahidi, Himani Sharma, Rajesh Gupta, Deepak Malhotra, Lance Dworkin, Steven Haller, David Kennedy

**Affiliations:** Department of Medicine, College of Medicine and Life Sciences, University of Toledo, Health Education Building RM 205, 3000 Arlington Ave, Toledo, OH 43614, USA; armelle.deriso@rockets.utoledo.edu (A.D.); mitra.patel@utoledo.edu (M.P.); dhanushya.battepati@rockets.utoledo.edu (D.B.); bella.khatibshahidi@rockets.utoledo.edu (B.K.-S.); himani.sharma@utoledo.edu (H.S.); rajesh.gupta@utoledo.edu (R.G.); deepak.malhotra@utoledo.edu (D.M.); lance.dworkin@utoledo.edu (L.D.); steven.haller@utoledo.edu (S.H.)

**Keywords:** vascular calcification, chronic kidney disease, CKD, uremic toxins, hyperphosphatemia, vascular smooth muscle cells, VSMCs, macrophages, endothelium

## Abstract

Vascular calcification (VC) is one of the major causes of cardiovascular morbidity and mortality in patients with chronic kidney disease (CKD). VC is a complex process expressing similarity to bone metabolism in onset and progression. VC in CKD is promoted by various factors not limited to hyperphosphatemia, Ca/Pi imbalance, uremic toxins, chronic inflammation, oxidative stress, and activation of multiple signaling pathways in different cell types, including vascular smooth muscle cells (VSMCs), macrophages, and endothelial cells. In the current review, we provide an in-depth analysis of the various kinds of VC, the clinical significance and available therapies, significant contributions from multiple cell types, and the associated cellular and molecular mechanisms for the VC process in the setting of CKD. Thus, we seek to highlight the key factors and cell types driving the pathology of VC in CKD in order to assist in the identification of preventative, diagnostic, and therapeutic strategies for patients burdened with this disease.

## 1. Introduction

Vascular calcification (VC) is a convoluted process that leads to pathological accumulation of calcium phosphate crystals in the intima and media layers of the vessel wall that worsens the course of atherosclerosis, diabetes, and chronic kidney disease (CKD) [1]. These mineral-enriched plaques induce arterial stiffening, putting patients at risk for fibrosis, inflammation, and oxidative stress on a cellular level. From a clinical perspective, VC is a major problem and is associated with worse outcomes in treatment of coronary artery disease (CAD) and peripheral artery disease (PAD). Coronary calcification is associated with worse outcomes after percutaneous coronary intervention (PCI), and VC is associated with higher risk of amputation after revascularization for lower extremity PAD [2,3]. These associations of CKD can directly increase risks of many clinical complications of VC such as worsening atherosclerosis and increased risk of vascular events such as myocardial infarction, stroke, and vascular occlusive events, which makes it a significant predictor of cardiovascular disease (CVD) and CKD [4,5,6,7,8]. We must give our attention to these patients in the clinical setting to detect early signs of VC in CKD and prepare for prevention and treatment.

VC is now considered as an active and finely regulated process similar to osteogenesis that involves cell-mediated processes and complex interaction between the inhibitor and promoter factors of the calcification process [9,10]. Table 1 lists some major promoter and inhibitor factors involved in VC.

Moderate to severe calcification manifests in the aorta, cardiac valves, and peripheral vessels, including the tunica intima and tunica medial layers. Intimal calcification is linked to atherosclerosis, while both intimal and medial calcification has been observed in CKD patients [8,11,12].

The pathogenesis of this complication involves an excess build-up of calcium deposits by active and passive means, a surplus in the excretion of osteoid matrix when cells are triggered by toxic stimuli, or an integrated pathway of both processes [13]. These pathways may be attributed to underlying mechanisms at the cellular level, such as iron, calcium, and phosphate metabolism dysfunction. Multiple key processes that attack metabolism regulation include hyperphosphatemia, calcium phosphate imbalances, and a surge in reactive oxidation species (ROS) due to iron misdistribution [13]. Environmental stimuli can trigger these imbalances, transforming vascular smooth muscle cells (VSMCs) into osteoblast-like cells. This results in the build-up of hydroxyapatite in the various layers of the major and minor vessels, inducing calcification in vascular cells [13,14].

Other mechanisms contributing to VC are related to high glucose levels, lipids, and low-density lipoproteins circulating in the endothelial lining [15]. Research efforts demonstrate how a multitude of these combined actions link VC to CKD, a pathophysiological process that develops in response to irregular environmental stimuli [13].

**Figure 1:**
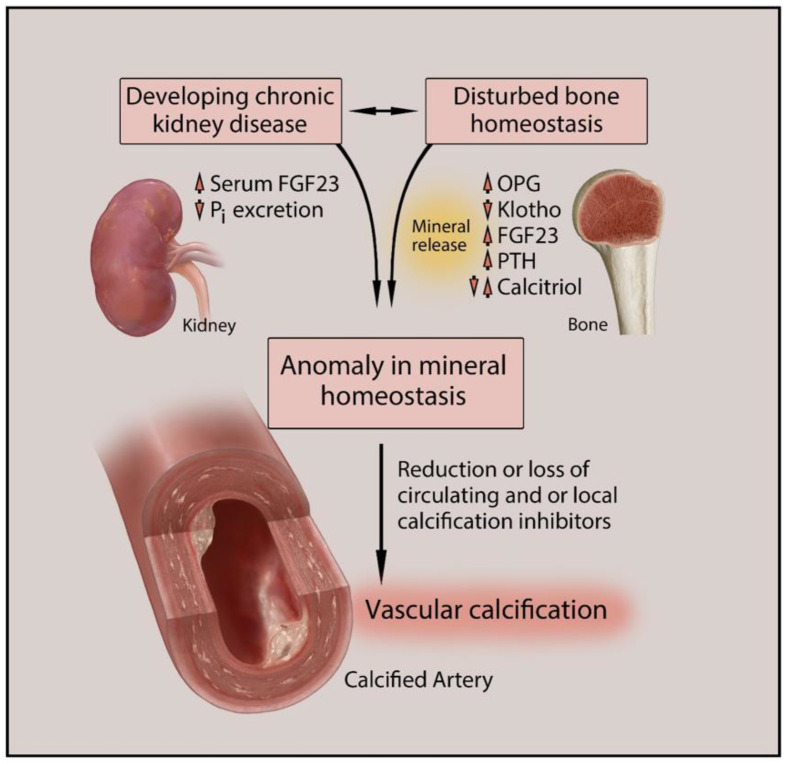
Pathogenesis of vascular calcification in chronic kidney disease (CKD). Altered bone metabolism and mineral homeostasis are commonly found and closely interconnected in CKD patients. The decline in kidney function also leads to elevated serum FGF23 levels and reduced inorganic phosphate excretion. The resulting pathological state is reflected by various altered biomarkers such as OPG, Klotho, PTH, and calcitriol. The disturbed mineral homeostasis also leads to altered serum and tissue levels of Ca, Pi, and Mg causing inflammation and other metabolic disorders. This leads to reduced or complete loss of circulating and/or local calcification inhibitors such as fetuin A, PPi, and MGP, causing vascular calcification. Ca, calcium; FGF23, fibroblast growth factor 23; Mg, magnesium; MGP, matrix Gla protein; OPG, osteoprotegerin; Pi, inorganic phosphate; PPi, inorganic pyrophosphate; PTH, parathyroid hormone. Upper arrow, increase; lower arrow, decrease.

Compared to the general population, patients with CKD are at an alarmingly higher risk of developing cardiovascular morbidity and mortality. About 50% of deaths from CKD are linked to CVD. Patients with chronic renal disease should be paid attention to for markers signaling cardiovascular complications [12,16]. Several studies show that cardiovascular calcifications in patients with renal disease are found to be more progressive and severe compared to non-CKD patients [6,13,17]. The pathogenesis of VC is enhanced in CKD through complex pathways. Modifications in iron, calcium, and phosphate levels due to kidney injury disturb the biochemical equilibrium, affecting bone remodeling in vascular cells [16]. Figure 1 depicts the pathogenesis of VC in CKD and its interconnection with altered bone and mineral homeostasis.

In the presence of risk factors such as VC, CKD patients are more likely to develop pulmonary hypertension (PH), an overlapping complication in patients with renal disease [18]. Additionally, the damaging effects of oxidative stress exacerbate VC in patients with CKD. Antioxidant defenses and free radical generation are balanced under homeostasis. When balance is disturbed, oxidative stress becomes a trigger of the cessation of cellular division. This becomes a marker of chronic and progressive diseases, including CKD and VC [16,19]. The prominence of VC in CKD can be attributed mainly to the combination of CKD traditional risk factors and the underlying uremic-specific mechanisms that induce the cardiovascular condition.

In this review, we discuss the major cell types involved in the VC process, the major cellular and molecular mechanisms involved in the disease, and the important diagnostic and therapeutic interventions that are used clinically to treat patients with VC.

## 2. Types and Anatomical Presence of VC

**Figure 2:**
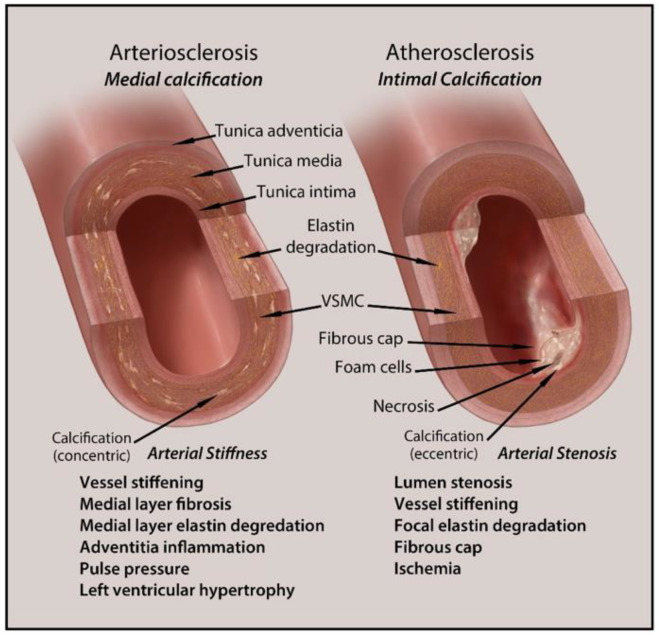
Schematic representation of intimal and medial calcification and their related pathologies.

VC pathogenesis shares similar mechanisms to that of bone formation. Both processes involve calcium deposition driven by bone matrix proteins, transcription factors, and stenosis. The two major classifications of VC are intimal and medial calcification. Figure 2 is a schematic representation of intimal and medial calcification and their associated pathologies.

A less common type of calcification involving calcium accumulation in arterioles is termed calciphylaxis. Intimal calcification shares a significant association with atherosclerosis, chronic inflammation, and the transformation of vascular smooth muscle cells (VSMCs) into osteoblast-like cells [8,20]. Medial calcification is more closely linked to elastin degradation, extracellular matrix remodeling events, and hyperphosphatemia. Diseases associated with this type include CKD, hypertension, and type 2 diabetes mellitus [20,21,22]. There is little known about the true cause of calciphylaxis, though the rare multifactorial syndrome has been found to have a close relationship with end-stage renal disease [23].

### 2.1. Intimal Calcification

Intimal calcification develops on the inner layer of vessel walls. This type shares a close link with disturbed lipid metabolism, making it a major indicator of atherosclerosis. Atherosclerosis results from excess lipoprotein depositions in the vessels sub-endothelial walls, resulting in the eccentric obstruction of lumen formation and matrix remodeling activities [20]. Calcification in atherosclerosis is driven by the osteochondrogenic process, which is initially induced by overexpression or inhibition of specific bone-related regulatory factors [7]. Dysfunction in the molecular signaling pathway during regulatory processes contributes to intimal calcification. A core element of the pathogenesis of this disease is inflammation. The osteogenic differentiation of VSMCs is promoted by the stimulus of atherogenic factors, including infiltration of inflammatory cells such as macrophages and recruitment of oxidized low-density lipoproteins and inflammatory oxylipids. These inflammatory factors are abundantly present in calcified vessels and therefore enhance the osteogenic process of VSMCs to osteoblast-like cells [20,21]. The severity and progression of VC can be attributed to the overactivity of inflammatory cytokines at the disease site. Chronic inflammation is linked to lipid accumulation at calcification sites, providing a clear association between the pathologies [7].

Also expressed in atherosclerotic plaques are bone matrix regulatory proteins and transcription factors such as bone morphogenetic protein (BMP) 2, osteopontin (OPN), osteocalcin (OC), and runt-related transcription factor 2 (RunX2). These regulatory factors function in the nucleation of hydroxyapatite minerals, found in plaques as calcified crystals [11,20]. BMP2 activity is strongly linked to VC development, as the protein is involved in the trans-differentiation of VSMCs into osteoblast-like cells, production of ROS, and inflammation. Elevated levels of dephosphorylated OPN have excitatory effects on VC as it can no longer inhibit calcium mineralization. When upregulated, master transcription factor RunX2 induces the transition of VSMCs into osteoblast-like cells during the osteogenic process. The mineralization of matrix vesicles further enhances osteogenic differentiation due to the metaplastic activity of calcifying vascular cells (CVCs) and VSMCs into chondral sites [11,20,21].

Intimal calcification in the aortic valve serves the highest mortality when in conjunction with end-stage renal failure. Atherosclerosis in the setting of renal insufficiency exacerbates the condition in a vicious cycle of calcific plaque development from excess calcium and phosphate precipitates [5]. Other conditions associated with the development of atherosclerotic intimal calcification include hyperlipidemia, hypertension, and osteoporosis [7,21].

### 2.2. Medial Calcification

The more extensive form of calcification in patients is medial artery calcification. It is significantly associated with cardiovascular complications such as diabetic arteriosclerosis and accounts for significant cardiovascular mortality [7]. Medial calcific sclerosis is a concentric process that develops in the medial layer of vascular walls and is more severe in type 2 diabetes mellitus. Several underlying mechanisms contribute to medial calcification, many of which are similar to the development of intimal VC. An isolated feature of medial calcification involves the degradation of the elastin-rich matrix [20]. Elastin, an extracellular matrix component, is the most abundant protein found in aortic walls. This protein serves the mechanical and elastic integrity of vessel walls, as they are subject to stress from high arterial blood pressure [22]. The formation of calcified vascular crystals obstructs the elastin fibers of arteries and vessels. Loss of this major extracellular constituent incites atherosclerosis, plaque rupture, and osteogenic differentiation [20,22]. The obstruction and destruction of elastin fibers predispose stenosis, ventricular hypertrophy, Marfan syndrome, and other progressive and fatal cardiovascular conditions [20,21].

Like intimal calcification, medial calcification is related to bone-related regulatory proteins and transcription factors, including BMP2, bone alkaline phosphatase (ALP), RunX2, and Msh Homeobox 2 (MSX2). BMP2 activity induces the transformation of smooth muscle cells in the medial layer to osteoblast-like cells. In arterial walls, upregulations in ALP matrix vesicles contribute to elastin degradation [20,21]. Activation of osteogenic transcription factors RunX2, MSX2, and ALP suppresses the activity of calcification inhibitors and promotes extracellular matrix remodeling and release of extracellular vesicles. With reduced levels of calcification inhibition, calcium and phosphate minerals accumulate, accelerating calcific plaque build-up and hyperphosphatemia [11,20]. Exceedingly high levels of circulating inorganic phosphate (Pi) are found to induce apoptosis in VSMCs. Calcium phosphate mineralization is then induced at the sites of smooth muscle cell apoptotic bodies, driving medial artery calcification [20].

Research findings suggest that the molecular mechanism behind hyperphosphatemia contributes to renal insufficiency. In one study using rat models with chronic renal failure, osteogenic factors and cartilage matrix were present in the aorta media at the site of calcified plaques [24]. The calcification process mirrors endochondral bone development, linking medial calcification to renal insufficiency disorders such as CKD [7,25].

### 2.3. Calciphylaxis

The rarest and most fatal form of VC is calciphylaxis or calcific uremic arteriolopathy. This progressive syndrome leads to extensive tissue and vessel necrosis, thrombosis, and sepsis. Calciphylaxis is found mostly in dialysis patients with end-stage renal disease by means of infection, prompting the high mortality rates [24,26]. Though this rare syndrome’s precise etiology is unknown, the condition has been linked to medial calcification, intimal hyperplasia, and other defects involving the calcium phosphate metabolic pathways [20]. Traditional risk factors and comorbidities also play a key role in the pathogenesis of this multifactorial disease. These include smoking, female sex, use of vitamin K antagonists, and overdose on warfarin agents. Comorbidities associated with cardiovascular and renal complications from calcification have been linked to calciphylaxis, such as diabetes mellitus, obesity, hyper- and hypoparathyroidism, hyperphosphatemia, and hypertension, chronic inflammation, and CKD [20,24,26].

Overall, the two major and prevailing calcification types share diverse similarities. Both intimal and medial calcification are involved in the osteochondrogenic process, beginning with calcium deposition at the site of vascular smooth muscle cells [20]. Vessel stiffening is also a common feature of both categories of calcification, whether induced by atherosclerotic intimal plaque development or medial crystal mineralization. The molecular signaling pathways shared by both involve regulatory proteins and transcription factors such as BMP2 and RunX2, each of which drives the osteogenic process [20,21].

On the other hand, calciphylaxis is less commonly found and not as well understood. Diseases such as diabetes, obesity and end-stage renal disease exacerbate it, bringing forth commonalities in its pathogenesis with intimal and medial calcification [26].

## 3. Major Cell Types of Vascular Calcification

### 3.1. Vascular Smooth Muscle Cells (VSMCs)

As we already know, VC is an actively driven process that is regulated by VSMCs. VSMCs have a contractile functionality responding to multiple signals such as acetylcholine and norepinephrine to express their phenotype. However, unlike other muscle cells, VSMCs do not terminally differentiate and exhibit phenotypic plasticity [27]. This allows them to vary their phenotypic expression based on environmental contexts, such as the various insults found in CKD. Initially, it was thought that once the initial insult was resolved, the VSMCs returned to their original contractile phenotype; however, recent findings suggest that they can continue to express a spectrum of phenotypes [28]. These phenotypes include osteochondrogenic, adipocytic, and foam cell deposition.

In CKD specifically, there are multiple active triggers that activate the calcification of primarily the medial layer of the vasculature, including inflammatory cytokines, uremic toxins, hypercalcemia, and hyperphosphatemia [29,30]. CKD primarily stimulates an osteochondrogenic phenotypic change in VSMCs. CKD is a chronic inflammatory state, and the accumulation of reactive oxygen species and inflammatory cytokines in the vasculature can precipitate RUNX2 and BMP-2 expression, which are known to stimulate osteocytic VSMC expression in the intima and media leading to VC [31,32,33]. They are normally balanced out by anti-calcification markers such as MGP, a BMP-2 inhibitor expressed in VSMCs, which are specifically known to be inhibited in CKD. Loss of these anti-calcification inhibitors in CKD has been shown to increase calcification in the vascular media [33,34]. Dysregulation of mineral homeostasis and hyperphosphatemia are considered the leading determinants of VC in CKD [35,36]. Calcium and phosphate both stimulate an osteochondrogenic phenotypic change in VSMCs individually and synergistically. Calcium is a key signaling mediator for VSMCs at physiologic levels [37]. Though the exact mechanism of the effect of hypercalcemia on VSMCs is unknown, calcium induces oxidative stress and can directly deposit in the vasculature. Extracellular calcium deposition can also decrease anti-calcification markers such as MGP, leading to increased calcium deposition. VSMCs express a calcium receptor (CaR), which regulates vascular tone. In vitro, calcified tissue had downregulated expression of this receptor, and complete removal in vitro from VSMCs significantly increased VC [35,37,38]. Mineral cellular phosphate transport is mediated by sodium-dependent phosphate cotransporters PiT-1 and PiT-2 in VSMCs [39]. Knockdown of PiT-1 has also been shown to decrease osteogenic marker expression such as RUNX2 in VSMCs and decrease calcification.

VSMCs not only have an osteochondrogenic phenotypic expression but can differentiate into foam cell-like macrophages, which deposit primarily in the vascular intima. VSMCs were found to be a contributing factor in many atheroma formations in the coronary arteries accounting for high levels of CD48 cells [40]. The differentiation of this phenotypic expression is understudied in CKD patients. However, the suspected trigger is increased oxidative stress as oxidation by reactive oxygen species is an early-stage contributor to atheroma formation [40].

VC in CKD is associated with increased deposition of VSCM-associated extracellular vesicles, which include matrix vesicles and apoptotic bodies [41,42,43]. Normally, contractile VSMCs release matrix vesicles to maintain homeostasis; however, in pathologic environments such as CKD (hyperphosphatemia, oxidative stress, inflammation), they can transform into a synthetic phenotype and increase secretion of matrix vesicles [44]. These transform target cells into calcified states, which then aggregate to form microcalcifications. They also decrease the expression of calcification inhibitors such as MGP and fetuin-A, like the osteogenic phenotypes [45].

**Figure 3:**
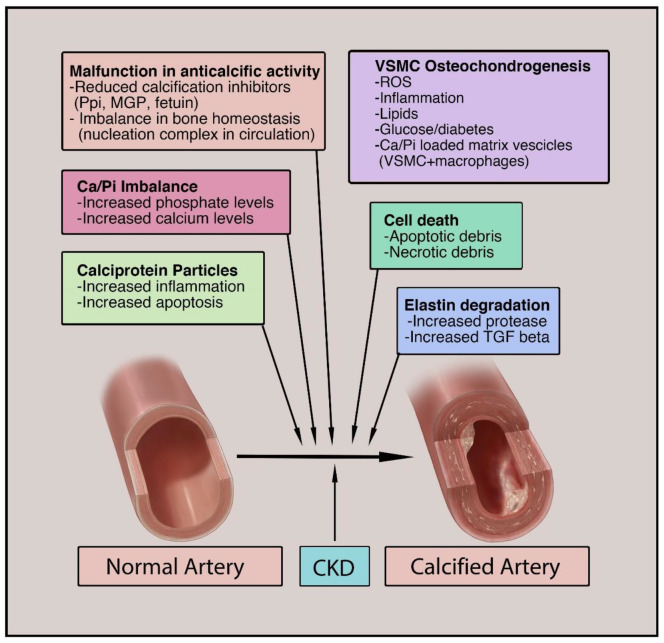
Major mechanisms involved in vascular calcification: Vascular calcification is an active process with multifactorial origins. Significant contributors to this include failure in the anti-calcification process, imbalance in bone homeostasis, osteochondrogenesis of VSMCs, calciprotein particles, cell death, elastin degradation, and extracellular vesicle formation and release. The combination of these factors in the setting of CKD leads to the development of both intimal and medial calcification. VSMC, vascular smooth muscle cell; Ca, calcium; Pi, inorganic phosphate; Ppi, inorganic pyrophosphate; MGP, matrix Gla protein; TGF, transforming growth factor; ROS, reactive oxygen species.

Along with phenotypic changes, VSMC apoptosis and autophagy play a significant role in VC in CKD. Due to increased oxidative and uremic stress in CKD, there is a significant increase in VSMC apoptosis. Apoptotic bodies have significantly high calcium concentrations. Upon apoptosis or necrosis of VSMCs, these vesicles release calcium and DNA, which ultimately deposit on the ECM of the vascular media leading to extensive calcification. Extracellular DNA has been shown to precipitate calcium and phosphate, which may contribute to arterial calcification. This is especially prominent in CKD or ESRD patients undergoing hemodialysis, as it can lead to increased VSMC apoptosis/necrosis through direct membrane contact and complement activation [46]. In normal physiologic conditions, low basal rates of autophagy are required for the removal of unwanted metabolites from the circulation [47]. The increased mineral levels and stress in CKD upregulate these rates and degrade the proteins necessary to maintain the contractile phenotype. This leads to the differentiation of phenotype along with direct calcific deposition in the vasculature [48]. A summary of the major mechanisms involved in VC in CKD is presented in Figure 3, with VSMC-associated osteochondrogenesis playing a central role in the process.

### 3.2. Macrophages

Monocytes and monocyte-derived macrophages are the crux of our innate immune system. They perform numerous tasks, including host defense, immune regulation, and tissue repair/regeneration [49]. Macrophages secrete many inflammatory substances such as TNF-a, IL-6, and IL-1b. These factors yield several processes that contribute to VC. Firstly, the factors lead to the differentiation of vascular wall cells into chondrocytes and osteoblasts. They can also stimulate the expression of bone morphogenetic protein and increase oxygen free radical production [50]. Calcium burden within the coronary artery is also positively correlated with IL-1b.

There are different subsets of macrophages that have unique roles in the process of calcification. The two most common subsets that are of interest to investigators are the M1, or “classical activated macrophage”, and M2, or “alternative activated macrophage”. The M1 type is activated by bacterial lipopolysaccharides and interferon-γ (IFN-γ). These macrophages typically produce reactive oxygen species (ROS), pro-inflammatory cytokines, and reactive nitrogen groups [51]. In certain conditions, and stimuli in various microenvironments, M1 and M2 macrophages display considerable plasticity and can trasnform between one phenotype and another [52]. M1 macrophages can directly release oncostatin M (OSM), which is a cytokine that belongs to the interleukin 6 cytokine group and stimulates vascular smooth muscle cells to take on an osteoblastic phenotype via the JAK-3-STAT3 pathway [53]. This subset of macrophages is also responsible for maintaining a persistent state of chronic inflammation that can interfere with the normal mechanism of VSMCs to differentiating into osteoblasts, leading to interspersed areas of fragmented calcification [54]. This chronic state of inflammation maintained by the M1 macrophages is associated with high levels of TNF-α and IL-6, suggesting that a causal link between macrophage-mediated inflammation and cardiovascular calcification exists in the setting of CKD [55]. Cartilage oligomeric matrix protein (COMP) is an interesting regulator as it facilitates macrophage polarity via integrin β3, which is a COMP-binding protein present on the surface of macrophages. It is known that COMP deficiency results in macrophages transforming into the M1 phenotype (osteogenic phenotype) and at the same time inhibiting macrophages from exhibiting the M2 phenotype, i.e., osteoclast-like cells [56].

The M2 phenotype is activated by Th2 cytokines, and these macrophages have an anti-inflammatory effect. They are involved in parasitic infection resistance, lipid metabolism, allergic reactions, and tumor progression [57]. Ricardo et al. studied how M2 macrophages are able to attenuate VSMCs from differentiating into osteogenic cells via the use of co-cultures of macrophages and smooth muscle cells where they are not in direct contact with each other [58]. Ricardo et al. also demonstrated that the inhibitory effect of M2 is related to increased secretion of adenosine triphosphate (ATP) secretion and the synthesis of pyrophosphoric acid (PPi) via fatty acid β-oxidation [58]. Another study illustrates that extracellular PPi functions as an endogenous inhibitor of vascular calcification both in vivo and in vitro [59]. PPi is generated from extracellular ATP by ectonucleotide pyrophosphatase/phosphodiesterase 1 (ENPP1), and a study by Johnson et al. shows that deleting ENPPI yields aortic calcification in mice [60].

Other functional substances secreted by macrophages include osteogenic genes such as tissue nonspecific alkaline phosphatase (TNAP), osteoprotegerin (OPN), and runt-related transcription factor 2 (Runx2), which further promote the osteogenic process [61]. Additionally, a recent study by Dube et al. showcased that the M1 subtype of macrophages differentiated from mice bone marrow-derived macrophage (BMDM) exhibits osteogenic properties via the constitutive activation of BMP-2 signaling [62]. Dube and colleagues have also discovered that deleting transient potential classical receptor 3 (TRPC3), a nonselective calcium channel in macrophages, leads to a reduction in apoptosis of macrophages triggered by endoplasmic reticulum stress and downregulates the expression of Runx2 and BMP2, leading to a reduction in calcification in advanced atherosclerotic plaques [63,64,65].

The formation of Ca/P nanocrystals in the vessels as a result of CKD stimulates macrophages to secrete pro-inflammatory cytokines, which in turn exacerbates VC [66]. In addition to the secretion of pro-inflammatory markers, high concentrations of Ca/P also trigger macrophages to release matrix vesicles (MVs). The MVs that are released have a proteomic profile similar to the MVs released by bone osteoblasts [67]. In the early stages of the development of VC, macrophages release an abundance of calcifying matrix vesicles (MVs), which contain the phosphatidylserine-annexin V-S100A9 complex [68]. Sophie et al. further studied this and confirmed that phosphatidylserine forms complexes with annexin V and S100A9 on the surface of macrophage-derived MV membranes. This complex then allows the entrance of calcium ions into the vesicles. The calcium and phosphate ions that enter the MVs via phosphate channels form calcium phosphorus complexes, which accumulate, forming the initial hydroxyapatite crystals [61]. These hydroxyapatite crystals continue to grow, rupturing the membrane, and keep growing to form calcified nodules. Studies have shown that the pro-inflammatory mediators mentioned earlier, such as IL-6, TNF-a, IL-1b, and oncostatin M, are not only involved in the osteochondrogenic transition of vascular/valvular cells but also the release of calcifying MVs [69].

The accumulation of uremic toxins in the circulation of CKD patients is the main driver of VC through the recruitment of monocytes and modulation of cells’ inflammatory capabilities [70]. The high levels of uremic toxins lead to the development of atherosclerotic calcification and Monckeberg’s medial calcification. There are two notable uremic toxins: indoxyl sulfate (IS) and paracresyl sulfate (pCS). IS and pCS are correlated with the presence of inflammatory markers such as TNF-a, IL-6, and IL-1B in CKD patients [71,72].

Experiments by Six et al. determined a high expression of adhesion molecules such as VCAM-1 and ICAM-1 when CKD mice were fed a high-phosphate diet [73]. This study essentially suggests when the expression of adhesion molecules by endothelial cells and monocytes is promoted, exposure to uremic toxins such as phosphate and IS leads to monocyte extravasation into cardiovascular tissues and thus inflammation-induced VC [70]. Evidence from several experimental studies suggest phosphate (Pi)/IS-induced monocyte recruitment, inflammation, lipid accumulation, and fibrosis are the major drivers of atherosclerotic plaque development and calcific aortic valve disease in CKD patients [70]. On the other hand, IS can also stimulate monocytes and macrophages to differentiate into the M2 phenotype [74]. The M2 phenotype leads to the expression of markers such as IL-10 and TGF-B, which yield an increase in profibrotic inflammatory macrophages [70]. The M2 macrophages demonstrate anti-calcific properties, which suggests that there is a compensatory mechanism. This mechanism protects tissues from developing pathologic calcifications associated with elevated serum phosphate levels [74].

pCS is another uremic toxin that is produced by intestinal bacteria. Various studies suggest that there is an association between increased pCS levels and renal function deterioration, atherosclerosis, and inflammation [70]. Experiments by Jing and colleagues demonstrate that when endothelial cells and macrophages are cultured with pCS in vitro, there is an increase in expression of inflammatory factors such as TNF-a and MCP-1, adhesion molecules [75]. Their studies show that the high levels of pCS seen in CKD patients could potentially be responsible for medial and intimal calcification [70]. The production of uric acid (UA) comes from the metabolism of nucleotides and ATP. It is the end product of purine metabolism [70,76]. It is seen that in patients undergoing hemodialysis, intimal and medial calcification are independently associated with blood uric acid levels. Geraci et al. suggest that in non-CKD patients with asymptomatic hyperuricemia, elevated serum UA levels are associated with carotid-intima media thickness [77]. The high UA levels are also associated with coronary artery calcification [78,79,80]. Nevertheless, the exact mechanism of how hyperuricemia leads to VC is unclear.

In supporting the findings of UA’s effects on macrophages, one study outlined the effects of allopurinol in reducing vascular inflammation. Allopurinol is a xanthine oxidase inhibitor used in treating gout and uric acid kidney stones. Xanthine oxidase is the enzyme responsible for the production of uric acid during purine metabolism. A study in mice showed that administering allopurinol led to reduced arterial stiffness, macrophage accumulation, decrease in vascular oxidative stress, and macrophage polarization to M1 [81]. This study outlines the integral role that UA plays in vascular inflammation, even though the exact causal link is yet to be determined. One key finding from Andrews and colleagues shows that UA may have an even more significant role in VC progression as the administration of allopurinol among stage 3 CKD patients did not impact the carotid-intima media thickness [82]. Among the many roles monocytes and macrophages are involved in, the complex array of phenotypes these cells exhibit is remarkable, especially in the development of VC in the context of CKD. These macrophages secrete many pro-inflammatory markers as outlined, differentiate into osteoclast-like cells, and also demonstrate protective mechanisms against inflammation and calcification. From the supporting studies and experiments, it is evident that the intermediate monocyte subtype plays a key role in the phenomenon of chronic inflammation leading to VC in patients with CKD [83]. Regardless of the many data and experiments that suggest the role of uremic toxins in promoting monocyte/macrophages to exhibit procalcific properties, the exact mechanism by which this event occurs is still unknown. Understanding the role of macrophages in the development of VC in CKD patients is critical. Given the diverse properties these cells exhibit in promoting this chronic disease, it is critical to understand the mechanisms whereby macrophages contribute to the calcification process for future studies regarding drug targets and treatments.

### 3.3. Endothelial Cells

**Figure 4:**
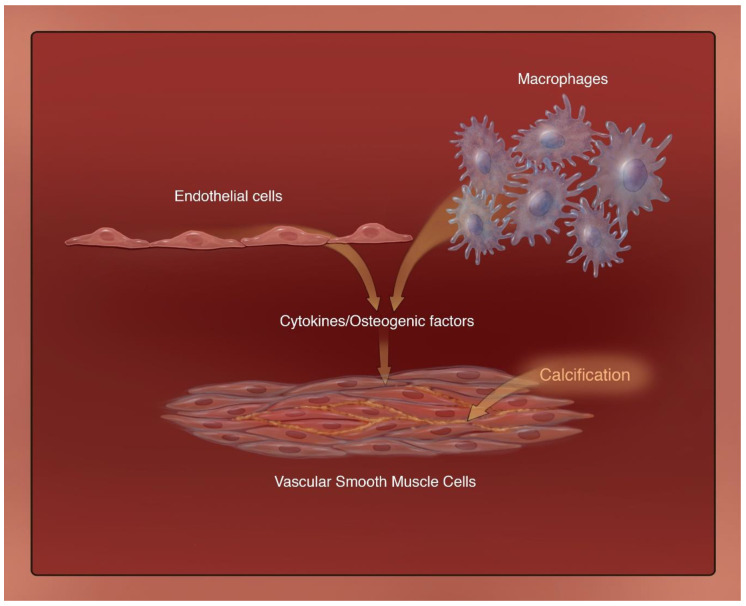
The cellular trifecta of vascular calcification: Endothelial cells and macrophages can secrete various cytokines and osteogenic factors that lead to the activation and progression of calcification in vascular smooth muscle cells.

The development of vascular pathogenesis is largely facilitated by the stability of the endothelium. Lack of integrity in this structure is a driving force of many vascular diseases, including VC [7,84]. Studies show that endothelial cells secrete soluble factors that play a key role in the calcification of the VSMCs [85,86,87]. The endothelial progenitor cells like macrophages are also known to be involved in the VC in CKD by expressing osteogenic factor osteocalcin [88]. Figure 4 highlights a central role of cytokines and osteogenic factors produced by both endothelial cells and macrophages that leads to calcification in VSMCs.

Endothelial cells also have the capacity to transform into mesenchymal stem cells to attain multipotency before they can differentiate into various cell types. This transformation is known as endothelial–mesenchymal transition (EndMT) [89]. EndMT is a vital mechanism for endothelial cells to undergo osteo/chondrogenesis and secrete factors involved in VC [87,90,91]. Studies by Min Wu et al. show that the use of calcimimetic suppresses the upregulation of mesenchymal markers and the downregulation of the endothelial marker, leading to reduced VC in the aortic samples of uremic rats [92].

Shear and mechanical stress are other factors that disrupt the endothelium and lead to worsening of VC. Vascular wall mechanical stress may contribute to endothelial and interstitial cell proliferation, altering the expression of calcification-related genes in cultured endothelial cells and fibroblasts [93]. Mechanical stress triggers the differentiation of preosteoblasts into mature osteoblasts and can drive the progenitors down the osteogenic lineage [94]. Mechanical stretch can upregulate the pro-osteogenic factors BMP-2 and Sprouty-1 [93,95], and both of these factors modulate the basal expression of osteogenic factors in untreated vascular fibroblasts. Additionally, the application of steady tension to fibroblasts results in downregulation of anti-calcification factors periostin and osteopontin [96,97]. Furthermore, mechanical stress leads to elevation of osteogenic genes thrombospondin and BMP-2 as well as other calcification-related genes [92,97].

Vascular endothelial cells are exposed to shear stress induced from patterns of blood flow [92]. Pulsatile endothelial shear stress can be very pronounced depending on the location and shape of the vessel [98]. This contributes to very turbulent blood flow and shear stress, where bifurcations in arterial tress are the most critical areas for calcification [99,100]. However, disrupted blood flow, with irregular flow and recirculation, can contribute to the development of atherosclerosis [94,99]. The hemodynamic forces, like fluid shear stretch and hydrostatic pressure, caused by pulsatile blood flow and pressure, cause significant stress on endothelial cells [94,100]. Shear stress is a critical modulator of endothelial phenotypes, activating mechanosensors on the endothelial cytoskeleton, which triggers the phosphorylation and activation of genes leading to increased endothelial distress [94]. Furthermore, the stress-induced expression of factors that leads to proinflammatory, procoagulant, proliferative, and proapoptotic functions can contribute to worsening atherosclerosis and VC [94].

Specifically, in CKD uremic vascular calcification occurs in part due to endothelial dysfunction. In a study by Yi-Chou Hou et al., vitamin D is discussed as having a preventative role in VC, especially for those with CKD. Vitamin D deficiency is common in CKD, in part due to proteinuria, the lower therapeutic active dose of vitamin D, and the decline of the glomerular filtration rate [101]. Supplementation of vitamin D has been shown to improve the dysfunction of the endothelium and protect against VC. The effect of vitamin D supplementation has on the endothelium in the vasculature is related to its suppression of the renin–angiotensin–aldosterone system (RAAS), decreased insulin resistance, decreased cholesterol via fewer foam cells, and vascular regeneration [101]. Interestingly, this study suggested that coronary artery calcification progression in those with diabetes mellitus and preserved renal function can be estimated with microalbuminuria.

Hyperphosphatemia, another condition prevalent in CKD due to the high concentration of inorganic phosphate, suppresses the mammalian target of rapamycin (mTOR) signaling. This leads to an increase in the protective autophagic process for endothelial cells by counteracting the reactive-oxygen-species-induced VC [102]. LC3, a protein associated with the autophagosome process, was increased in the endothelial cells of the CKD rat model compared to the controls. During autophagy, LC3 positive vesicles join with ubiquitin and p62, autophagy adaptor. Next, the proteins combine with the lysosomal marker LAMP1 to complete the autophagy process. The end result is calcium and phosphate build-up, contributing to VC [103].

Furthermore, a study by Bouabdallah, et al. shows that Pi and IS together fostered the secretion of interleukin-8 (IL-8) from the endothelial cells of human aortic smooth muscle cells, which induced calcification. It was proposed that IL-8 blocked the production of the potent calcification inhibitor, osteopontin. When antibodies to IL-8 were used, this prevented aortic calcification [104].

Hypertension and VC usually occur together and are ultimately related to one another. Both diseases share endothelial dysfunction. Meng, et al. looked at the aortic calcification in hypertensive rats. They observed a correlation with the expression of the calcium-promoting proteins, MMP-2, and MMP-9 levels in endothelial cells and vascular smooth muscle cell calcification [105]. This is yet another example of endothelial cell dysfunction leading to VC.

In conclusion, endothelial damage leading to VC stems mainly from a variety of reasons, which cause oxidative stress and an increase in an inflammatory response to the endothelium. Crucial factors that lead to endothelial dysfunction include CKD, dyslipidemia, protein-bound uremic toxins, and hypertension.

## 4. Clinical Implications and Diagnosis of Vascular Calcification in CKD

Vascular disease is the most common cause of death in patients with CKD. In CKD, there are multiple contributing factors to increased VC, such as hyperphosphatemia, hypercalcemia, and increased levels of parathyroid hormone (PTH) [37,106]. High levels of these electrolytes and hormones cause increased cellular activity that increases matrix mineralization, deposition of hydroxyapatite, and inflammation in the vascular intimal and medial walls [107,108,109].

Amongst CKD patients, VC has been proven to increase cardiovascular mortality and morbidity; however, its predictive value is not clear. Additionally, the increased amount of VC increases one’s risk for heart failure, limb ischemia, stroke, and myocardial infarction. Because no data suggest that early detection benefits patients, we generally do not screen CKD patients for VC. Moreover, it is controversial whether interventions that delay the progression of calcification can clinically improve patient outcomes [110].

VC can be diagnosed, detected, and monitored through multiple modalities of imaging, including ultrasound, plain radiographs, and computed tomography (CT) scan. Ultrasound is usually limited to large superficial vessels such as the carotid and femoral arteries, but the information received is qualitative at best and not good for monitoring [111]. Plain radiographs have been used more often for the detection and quantitation of VC. This is normally seen when assessing larger arteries such as the aorta, femoral, and iliac arteries on both anteroposterior and lateral films. The limitation of this imaging modality is that it is difficult to measure VC progression over short periods of time and can be subjective based on the reader [111]. It is also semiquantitative and not as sensitive as a CT scan, which is a superior modality of assessment. VC is detected in >80% of patients undergoing dialysis, which are mostly late CKD and early end-stage renal disease (ESRD) patients. The prevalence amongst non-dialysis CKD patients is around 47% to 83% [110,112,113,114,115,116,117,118]. Multi-slice CT (MSCT) scans are often used for diagnosis and follow up for VC in CKD patients. The high image quality allows for precise, quantitative measurements of VC [110,111]. VC in CKD patients is known to be prominent in the media; however, none of these modalities can definitively differentiate intimal from medial calcification [111]. Currently, there are many controversies on the benefit of screening CKD patients for VC. Even if routine screening is performed, there is limited evidence that modifying risk factors and therapies have a significant impact on clinical outcomes [119]. The Kidney Disease: Improving Global Outcomes (KDIGO) guidelines do weakly recommend screening for VC in CKD patients with either lateral radiographs or CT scan [114,120].

## 5. Treatment Options for Vascular Calcification in CKD

Treatment options for VC in patients with chronic kidney disease include lifestyle modifications, pharmacologic agents, and surgical interventions. One of the most important lifestyle modifications that decreases mortality rates in these patients is smoking cessation [121].

Medical management in these patients is four-fold. Antiplatelet therapy to reduce platelet aggregation, blood pressure control, anti-lipid therapy for plaque-stabilization, and calcium/phosphate balance to prevent further calcification. Antiplatelet therapy, such as aspirin or clopidogrel, has been shown to reduce major cardiovascular events and overall mortality [122]. Blood pressure control is essential; however, there are no specific studies that show that aggressive blood pressure control has altered the course of peripheral artery disease in patients with CKD. Antilipid therapy such as statins significantly reduces the risk of major atherosclerotic events by stabilizing plaques from rupturing.

Though these three targets are important controls of peripheral vascular disease in CKD, calcium and phosphate balance is essential in preventing the progression of VC. Maintaining a calcium phosphate product of less than 60 is imperative in preventing the rapid progression of VC and complications such as calciphylaxis. Phosphate binders are recommended in all patients in CKD to lower phosphate levels [123]. There are two types of phosphate binders, calcium-based and non-calcium-based. Non-calcium-based phosphate binders such as sevelamer are more frequently used due to providing an all-cause mortality benefit in patients in CKD vs. calcium-based phosphate binders such as calcium acetate/magnesium carbonate [124]. Calcimimetics such as Cinacalcet act on calcium-sensing receptors providing a negative feedback loop to decrease PTH levels in an attempt to decrease serum calcium and phosphate levels. Though effective in reducing serum PTH levels, these medications have a questionable impact on all-cause mortality in patients with CKD [125]. Additional studies still need to be performed for assessment. Vitamin D supplements decrease PTH levels by a negative feedback mechanism. Vitamin D deficiency can cause endothelial dysfunction, which can worsen VC. Preventing this by supplementation can attenuate VC in CKD patients [126]. Currently, the KDIGO guidelines recommend vitamin D supplementation in all patients with CKD stage 4 and 5 [120].

Other options include osteoclast activity inhibitors such as bisphosphonates and denosumab. Bisphosphonates such as alendronate are antiresorptive drugs that inhibit osteoclast activity, preventing the release of calcium and phosphate into the bloodstream. They are usually well tolerated but bisphosphonates can cause worsening nephrotoxicity, focal segmental glomerulosclerosis, and osteonecrosis of the jaw [127,128]. Denosumab is a monoclonal antibody that inhibits NFkB-ligand (RANKL), which blocks its osteoclastic and resorptive properties. Few studies have been performed on the effect on VC in vivo and mortality benefits of both bisphosphonates and denosumab in CKD patients; therefore, their clinical benefit is still unclear, and they are not routinely recommended in these patients [123]. Other options also include magnesium supplementation, which has been shown to decrease phosphate-induced calcification in vitro; however, there are very few studies that have shown a statistically significant impact on reducing VC in CKD patients in vivo [123,129].

Interventions include revascularization or amputation. Revascularization via open surgical bypass or percutaneous angioplasty/stenting (also called endovascular revascularization) is usually used in patients experiencing acute limb ischemia or chronic claudication or critical limb ischemia with non-healing wounds. There are no clear data on which type of revascularization is more beneficial, but many physicians would prefer an endovascular first approach if the anatomy is amenable. Amputation is usually reserved for patients that fail revascularization or have non-viable limbs as suggested by symptoms such as paralysis, severe ulceration, or gangrene. Dialysis patients have an extremely high rate of nontraumatic lower extremity amputation compared with the general population [130]. As noted earlier, VC is associated with worse prognosis after coronary or peripheral vascular revascularization procedures [2,3].

There are few experimental therapies that are being studied to combat VC such as ethylenediaminetetraacetic acid (EDTA) chelation therapy, inducing a certain amount of metabolic acidosis, and the use of autologous osteoclasts [11]. EDTA is an amino acid that can bind calcium potentially decreasing free calcium in the blood. While a small human trial showed regression of VC in patients using EDTA, the study was confounded by the lack of an appropriate control group [131]. A state of metabolic acidosis can activate osteoclasts via RANKL and has been shown to decrease VC in vivo and in vitro; however, there are negative side effects of acidosis, and the risks vs. benefits have not been studied [132,133]. Lastly, localized osteoclast therapy from rat-derived bone marrow has been shown to decrease VC in vitro. This has promising potential as it can be used to treat or even prevent VC in certain areas; however, the clinical application of this approach is speculative [130,134]. Along these lines, there are many experimental treatments under active investigation, but more pre-clinical and clinical data will be necessary to determine the efficacy and safety of these treatments.

## 6. Animal Models of Vascular Calcification

Various animal models have been evaluated in the investigation of VC mechanisms and complications. These studies contribute to understanding the complications that lead to calcification in humans [135]. One review examined CKD rodent models from multiple trials, where the animals developed varying degrees of vascular calcification in the CKD setting. It was found that a particular rodent model shared strong similarities in the symptom development with CKD patients in the clinical setting [136]. This particular rat model developed CKD through the supplementation of adenine, a renal toxin, which was later found to cause nephrotoxicity as a result of 2,8-hydroxyadenine accumulation in the urinary tract [136]. This symptom overlaps with CKD development in patients, providing a useful animal model for CKD-related VC examinations [135]. Several rodent models present conditions including hyperparathyroidism, hyperphosphatemia, and increased levels of both blood urea nitrogen and plasma creatinine [136]. Differences in calcification severity were attributed to varying diet plans, genotypes, and level of kidney damage. As these CKD-related VC animal models share several similarities with the development of VC in CKD patients, they may help reveal important pre-clinical insights [135,136].

Induction of VC through the modification of calcium phosphate levels, alterations in lipid metabolism, downregulation of calcification inhibitors, and promotion of uremic conditions is another approach to modeling VC in CKD [137]. Following these modifications, the mechanisms of VSMCs in the mineralization of the vessel walls of the models is quite similar to the role of VSMCs in humans. These VSMCs are of particular importance as they are associated with VC across multiple species, thus underscoring the hallmark contribution of this cell type [135]. This point is highlighted by several studies where VSMCs have been demonstrated to play an essential role in VC induction. Inhibition of the matrix-gla protein (MGP) yielded substantial VC where VSMCs formed profound cartilage in vessels through osteogenic differentiation [137]. Deficiency in osteopontin (OPN) is another mediator of calcification that induces mineralization in mouse models [135,137].

Altogether, the results in VC animal models provide valuable insight into the mechanisms of VC development in humans [137]. The many factors of VC regulation are comparable across various species from mice to humans making these animal models appropriate for the study of this complex disease and allowing for the needed pre-clinical development of therapeutic interventions [138].

## 7. Conclusions

Altogether, an extensive multitude of associated factors relates VC to CKD. A wide range of comorbidities escalates VC pathogenesis, including hypertension, hyperglycemia, hyperphosphatemia, hyperlipidemia, inflammation, oxidative stress, and uremia. Structural factors such as the anatomical location of the affected vessel and histological sites of calcification also lead to the advancement of VC. There likewise exists a variety of molecular mechanisms that play essential roles in the pathogenesis of the vessel’s calcification. Conventional and alternative prevention mechanisms such as regular exercise, maintaining a healthy diet, and therapeutic approaches are opportunities to decrease the VC burden in CKD. However, there is limited research demonstrating that these mechanisms effectively improve the severity of VC. By defining the exact pathology of the various forms of VC in CKD, targeted and rationale strategies can be developed to understand the clinical importance and address the potential pathological sequalae in this at-risk population.

## Figures and Tables

**Table 1:** Major promoter and inhibitor factors involved in vascular calcification.

Promoters	Inhibitors
Bone morphogenetic protein (BMP) 2, 4, and 6 Osteocalcin Alkaline phosphatase (Sex determining region Y)-box 9 (SOX9) Osterix Matrix metalloproteinase (MMP) 2, 3, and 7 Runt-related transcription factor 2 (Runx2) Calcium Phosphate Glucose Advanced glycation end products Oxidized low-density lipoproteins Collagen I Receptor activator of nuclear factor-kB ligand (RANKL)	Matrix Gla protein (MGP) Osteopontin Osteoprotegerin Vitamin K Magnesium Bone morphogenetic protein 7 (BMP7) Fetuin Klotho Parathyroid hormone (PTH) Pyrophosphate Carbonic anhydrase Collagen IV Inorganic pyrophosphate (PPi)
